# Genetic associations with neural reward responsivity to food cues in children

**DOI:** 10.3389/fnut.2024.1387514

**Published:** 2024-09-25

**Authors:** Dabin Yeum, Timothy J. Renier, Delaina D. Carlson, Grace A. Ballarino, Reina K. Lansigan, Meghan L. Meyer, Ruth J. F. Loos, Jennifer A. Emond, Travis D. Masterson, Diane Gilbert-Diamond

**Affiliations:** ^1^Department of Epidemiology, Geisel School of Medicine at Dartmouth College, Lebanon, NH, United States; ^2^Department of Pediatrics, Geisel School of Medicine at Dartmouth College, Lebanon, NH, United States; ^3^Department of Psychology, Columbia University, New York, NY, United States; ^4^Novo Nordisk Foundation Center for Basic Metabolic Research, Faculty for Health and Medical Sciences, University of Copenhagen, Copenhagen, Denmark; ^5^Charles Bronfman Institute for Personalized Medicine, Icahn School of Medicine at Mount Sinai, New York, NY, United States; ^6^Department of Biomedical Data Science, Geisel School of Medicine at Dartmouth College, Lebanon, NH, United States; ^7^Department of Nutritional Sciences, College of Health and Human Development, The Pennsylvania State University, University Park, PA, United States; ^8^Department of Medicine, Geisel School of Medicine at Dartmouth College, Lebanon, NH, United States

**Keywords:** food cues, fMRI, polygenic risk score, children, genotype, reward

## Abstract

**Objective:**

To test associations of candidate obesity-related single nucleotide polymorphisms (SNPs) and obesity polygenic risk scores (PRS) with neural reward reactivity to food cues.

**Methods:**

After consuming a pre-load meal, 9–12-year-old children completed a functional magnetic resonance imaging (fMRI) paradigm with exposure to food and non-food commercials. Genetic exposures included *FTO* rs9939609, *MC4R* rs571312, and a pediatric-specific obesity PRS. A targeted region-of-interest (ROI) analysis for 7 bilateral reward regions and a whole-brain analysis were conducted. Independent associations between each genetic factor and reward responsivity to food cues in each ROI were evaluated using linear models.

**Results:**

Analyses included 151 children (*M* = 10.9 years). Each *FTO* rs9939609 obesity risk allele was related to a higher food-cue-related response in the right lateral hypothalamus after controlling for covariates including the current BMI *Z*-score *(p* < 0.01), however, the association did not remain significant after applying the multiple testing correction. *MC4R* rs571312 and the PRS were not related to heightened food-cue-related reward responsivity in any examined regions. The whole-brain analysis did not identify additional regions of food-cue-related response related to the examined genetic factors.

**Conclusion:**

Children genetically at risk for obesity, as indicated by the *FTO* genotype, may be predisposed to higher food-cue-related reward responsivity in the lateral hypothalamus in the sated state, which, in turn, could contribute to overconsumption.

**Clinical trial registration:**

https://clinicaltrials.gov/study/NCT03766191, identifier NCT03766191.

## 1 Introduction

Obesity currently affects approximately 20% of children and adolescents aged 2–19 years in the United States according to the National Health Statistics Report (1). Obesity often continues from childhood into adulthood (2), and obesity in adulthood is a known risk factor for the development of multiple comorbidities including type 2 diabetes, cardiovascular diseases, and cancer (3). The etiology of obesity is multifactorial, affected by genes, environment, and gene-environment interactions (4, 5).

Environmental food cues include the smell, taste, and sight of highly palatable food or food-related situations and play a crucial role in obesity development through physiological and psychological responses (6–9). They are often presented through media in the form of advertisements for highly palatable, nutrient-poor, and energy-dense foods and beverages (10). Marketing of highly palatable foods and beverages is often directed toward younger children, and these exposures promote the development of unhealthy food preferences and eating behavior patterns that may ultimately lead to childhood obesity (10–15).

Exposure to highly palatable food and beverage cues may activate a response in dopaminergic reward regions of the brain that are associated with increased food consumption (16–18) and weight gain (19–21). Previous functional magnetic resonance imaging (fMRI) studies have identified the brain regions involved in the corticolimbic reward circuitries related to food cues: nucleus accumbens, orbitofrontal cortex, amygdala, insula, lateral hypothalamus (LH), substantia nigra, and ventral tegmental area (22–28). Further, these regions have been related to food cravings and appetitive motivation (29–33).

Several genes, including the fat mass and obesity-associated gene (*FTO*) and melanocortin 4 receptor gene (*MC4R*), have been implicated in obesity risk. Research has shown that divergent central nervous system (CNS) mechanisms may drive overconsumption in those with *FTO* risk alleles. A rodent study has shown that *FTO* is present primarily in the hypothalamus, a region related to hunger/satiation control (34). A common genetic variant within the first intron of the *FTO* gene, rs9939609, is known to be associated with higher energy intake (35–38) and BMI (39–41) in children. A previous study from our group also found that *FTO* rs9939609 was associated with children’s food-cue-related neural reactivity in the left and right nucleus accumbens (42), a potential mediator of excess consumption and adiposity gain. Participants were only provided a light snack in that study to help control their hunger, so the association between *FTO* and food cue reactivity in the post-prandial state was not examined.

Melanocortin 4 receptors are also expressed in the hypothalamus as part of the leptin-melanocortin pathway and play a crucial role in regulating appetite, energy balance, and body weight (43–45). *MC4R* rs571312, a common near-*MC4R* variant, has been related to higher caloric intake (46) and greater BMI or obesity (47–49). Another study found a strong association between rs12970134 and obesogenic eating behaviors including greater food responsiveness and less satiety responsiveness in children (50). However, no studies to date have examined the effect of common *MC4R* polymorphisms on food-cue-related neural reward reactivity.

Though some individuals may have obesity due to a rare mutation in a single gene, most individuals with obesity have numerous polymorphisms that jointly affect their adiposity (51). A comprehensive genetic obesity risk can be summarized through an obesity polygenic risk score (PRS) that is constructed based on the effects of variants observed in genome-wide association studies (GWAS). Richardson et al. (52) created a 295 SNP PRS to predict adiposity in early life. Though previous studies in children have demonstrated an association between weight status and food-cue-related neural response to food cues (21, 53–55), the relationship between comprehensive genetic risk for obesity, characterized by a PRS, and food-cue-related neural responsivity has not yet been examined.

In this study, we aimed to test whether some children are genetically predisposed to heightened food-cue-related neural reward reactivity in the post-prandial period. We hypothesized that the genetic risk of obesity, defined by *FTO* rs9939609, *MC4R* rs571312, and a pediatric PRS, would relate to greater differential activation in brain reward regions in response to food advertisements. Additionally, we conducted a hypothesis-generating whole-brain exploratory analysis to identify additional regions that may be related to the associations between genetic exposures and post-prandial responsivity to food cues. This study builds upon previous work by examining a greater range of obesity-related factors and by examining how these genetic factors affect neural food cue reactivity when children are in the sated state.

## 2 Methods

### 2.1 Study participants

The data in this paper are from a larger study measuring the genetic associations with children’s neural reward reactivity and eating in the absence of hunger in response to food cues. The study enrolled 189 pre-adolescent children from the Northern New England community. Seven participants were excluded after genotyping quality control, and 31 participants were excluded after MRI scanning quality control. Scan data from 31 participants were excluded due to: refusal to be scanned (*n* = 12); excessive movement in the scanner (*n* = 9); and technical issues (*n* = 10). The final analysis included 151 children (86 of whom were male) between the ages of 9 and 12 [mean (SD) = 10.9 (1.16) y]. Dartmouth College’s Committee for the Protection of Human Subjects approved all study protocols.

### 2.2 Study overview

Participants attended a study visit alongside a parent or guardian. Visits were scheduled at either lunchtime (11:00 am–1:00 pm) or dinnertime (4:00 pm–6:00 pm). A trained research staff member collected children’s saliva samples for genetic analysis, measured height and weight, and administered questionnaires to the parents. The parent-reported child’s physical activity, date of birth, biological sex, race, ethnicity, annual household income, and parent education level. Participant height was measured to the nearest 0.1 cm using Seca 264 Stadiometer (Hamburg, Germany), and weight was measured to the nearest 0.01 kg using a Seca 703 Medical Scale (Hamburg, Germany). Children consumed a standardized pre-load meal consisting of macaroni and cheese, apple sauce, corn, milk, and water. Satiety level was measured prior to the scan using the Freddy Fullness scale (56), a validated visual analog scale for estimating fullness in children. The fullness scale was reported across a range of 0-150 mm and converted into percentages (out of 150 mm); higher scores indicated greater fullness.

### 2.3 Genotyping

DNA extracted from saliva samples was genotyped for >600,000 single nucleotide polymorphisms (SNPs) with the Illumina Global Screening Array 24 v1.0 or v3.0 (57). Pre-specified quality control thresholds were applied to generate genotype calls using GenomeStudio software (58) with downstream quality control steps and determination of European or non-European ancestry with principal components, as previously described (59, 60). Using the Michigan Imputation Server, haplotype-based imputation was performed with a quality score threshold of *R*^2^ > 0.8 selected for SNPs with high-quality imputation (61, 62). Seven children were excluded for failing genotype quality control.

As primary exposures of genetic risk, two single SNPs were considered (*FTO* rs9939609 and *MC4R* rs571312), and a pediatric-specific PRS with 265 of 295 SNPs available in the data (52), the “Pediatric PRS.” In additional exploratory analyses, we also analyzed three PRS previously associated with adult BMI. These included a 97 SNPs PRS (63), 557 SNP PRS (52), and a ~ 2 million SNP PRS (64), henceforth referred to as the “Adult 97 PRS,” “Adult 557 PRS,” and “Adult 2M PRS,” respectively. Each PRS was computed as the product of the dosage of each risk allele (0, 1, or 2) and the published effect size, summed and standardized into *Z*-scores.

### 2.4 Scanning paradigm

Using E-Prime (65), children were presented with a series of videos that were designed to replicate a typical television show. The stimuli included three 5 min segments of a popular science show (MythBusters) interspersed with four 5 min commercial breaks.

Four functional runs were conducted in each scan. Additional functional runs were collected as part of the larger study after the experimental paradigm of this study, but are not relevant to this presented analysis. Each functional run began and ended with a 15 s presentation of a fixation cross. For each run, 5 food and 5 non-food TV commercials were presented which alternated in an AB pattern (66, 67). The block pattern for each run was randomized between participants (AB or BA) and which commercials were played during each block were also randomized between participants. Each commercial ran for approximately 15 s, and each functional run was approximately 5 min in length. The total duration of the scan was approximately 1 h.

### 2.5 Stimuli

Age-appropriate food and non-food commercials that were included in this study were rated by children for interest and excitement (42). There was no overall difference in interest and excitement between the food and non-food commercials.

### 2.6 Image acquisition

Scans were conducted using a 3.0 T Siemens MAGNETOM Prisma MRI scanner equipped with a 32-channel head coil. For T1-weighted structural scans (MPRAGE), the following parameters were employed: echo time (TE) of 2.32 ms, repetition time (TR) of 2,300 ms, flip angle of 8 degrees, matrix size of 256 × 256 mm, field of view of 240 × 240 mm, 192 slices with a slice thickness of 0.9 mm, and voxel size of 0.9 × 0.9 × 0.9 mm. Functional imaging utilized a T2*-weighted echo planar imaging (EPI) sequence with TE = 33 ms, TR = 1,250 ms, flip angle = 64 degrees, matrix size = 96 × 96, field of view = 240 × 240 mm, 56 slices with a slice thickness = 2.5 mm, and voxel dimensions of 2.5 × 2.5 × 2.5 mm. Four functional runs of 144 volumes were included in the analysis for each participant.

### 2.7 Model covariates

BMI was calculated based on participants’ height and weight using the U.S. Center for Disease Control and Prevention (CDC) 2020 age-and sex-specific distributions (68). A missing value for physical activity (*N* = 1) was imputed with the most frequently reported value. A missing value for the fullness measure (*N* = 1) was imputed with the median value. One fullness value of 180 mm was imputed with the median due to researcher measurement error.

### 2.8 MRI pre-processing

Anatomical data preprocessing and functional data preprocessing were performed using *fMRIPrep* 1.2.5 (69, 70), which is based on *Nipype* 1.1.6 (71, 72). The pipeline and protocol used are described in detail as a template provided by *fMRIPrep* in a previously published article (73).

### 2.9 Statistical analysis

#### 2.9.1 Subject-level analysis

Following pre-processing, participants’ individual fMRI data were analyzed using the NLTools Python package (74). A general linear model (GLM) was conducted for subject-level analysis for each participant. This included constructing a design matrix, convolving with the hemodynamic response function (HRF), incorporating nuisance variables such as intercepts, linear and quadratic trends, motion covariates (comprising 24 parameters: six demeaned realignment parameters, their squares, derivatives, and squared derivatives), and identifying motion spikes (defined as spikes between successive TRs and global spikes exceeding an intensity change threshold of 2.5 standard deviations from the mean). The data underwent spatial smoothing using a Gaussian kernel with a full-width at half maximum (FWHM) of 6 mm. We conducted a standard visual inspection of scans with a frame-wise displacement of 1 or greater, and one of four functional runs was excluded for 17 participants (~11%) due to such visual inspections (73). Additionally, we examined functional runs for extreme head motion defined as >25% motion spikes (> 36 spikes) of the scan volumes; however, no functional run was excluded from further analyses. To generate the food-specific regression coefficient maps for individuals, the coefficients in each voxel were averaged across functional runs for food and non-food ad conditions, separately, and then the within-subject difference between the two conditions was computed to create contrast maps. All individual-level contrast maps were used in the targeted region of interest (ROI) and whole brain analyses. Multiple comparison correction using the false discovery rate (FDR) was applied to the *p*-values across 7 bilateral ROIs with significance set at *q* < 0.05.

#### 2.9.2 Region of interest analyses

**Figure 1:**
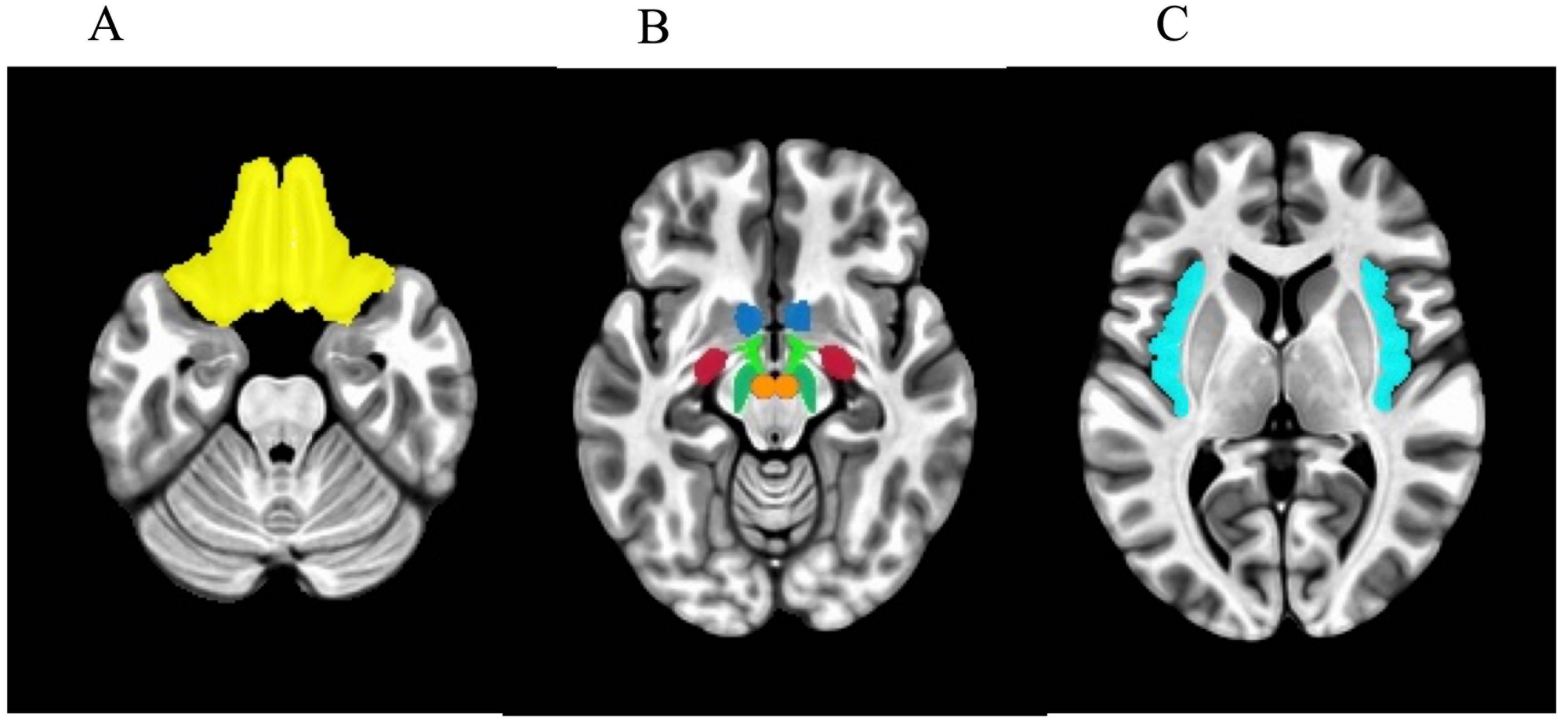
Axial view of masks used in the regions-of-interest (ROI) analysis. **(A)** Orbitofrontal cortex. **(B)** Blue: nucleus accumbens. Light green: lateral hypothalamus. Red: amygdala. Dark green: substantia nigra. Orange: ventral tegmental area. **(C)** Insula.

For the *a priori* ROI analysis, seven bilateral ROIs were selected as candidate reward regions based on previous literature (25): the nucleus accumbens (NAcc), orbitofrontal cortex (OFC), amygdala, insula, lateral hypothalamus (LH), ventral tegmental area (VTA), and substantia nigra (SN). The masks of the bilateral NAcc, OFC, amygdala, and insula were extracted for each participant using FreeSurfer’s autosegmentation.^1^ The final group-level ROI masks were created by including those voxels that are counted in the individual-level masks for at least 75% of participants. As FreeSurfer does not include autosegmentation of the lateral hypothalamus and SN, masks of these regions were generated using the anatomical atlas of the human hypothalamic regions (75). The mask of the ventral tegmental area was defined by the sphere with a radius of 5 mm centered at the MNI coordinate [±4, −16, −10] (76). The ROI masks are shown in Figure 1. Beta values were then averaged across each mask and analyzed using R (77). We investigated the Pearson correlation between corresponding lateral reward ROIs as well as across reward ROIs.

Child age, biological sex, BMI *Z*-score, satiety level post preload-meal, and European ancestry were selected as covariates *a priori* for all adjusted models given potential relationships with genetic exposures and/or neural response to stimuli, for all adjusted models. After examining the bivariate relationships between the other potential covariates and the neural response in any ROIs using a threshold of *p* < 0.1, physical activity and annual household income were added into adjusted models. Linear models were used to test the independent associations between the genetic factors (*MC4R* rs571312, *FTO* rs9939609, Pediatric PRS) and the neural response in the bilateral ROIs. In an exploratory analysis, the linear models were repeated with the secondary genetic exposures of 3 adult obesity PRS. Given that the pediatric and adult obesity PRS measures were trained on individuals of European ancestry, the distribution of each genotype and PRS in participants with European and non-European ancestry was explored in Supplementary Table S1, and the regression models of the four PRS measures were repeated restricting the sample to participants with European ancestry in a sensitivity analysis.

#### 2.9.3 Exploratory whole-brain analysis

A whole-brain analysis was conducted using the individual beta maps as input to a group-level analysis to test the unadjusted and adjusted linear relationships between food-cue-related response and genotypes. To determine significance at the group level, an initial voxel-wise significance threshold of *p*-value <0.001 was applied and was then cluster-corrected using a threshold of a cluster size of *k* = 90 for an overall *p*-value <0.05. Clustering parameters were based on 10,000 Monte-Carlo simulations determined using 3dClustSim from AFNI.

## 3 Results

Seven participants were excluded after genotype quality control, and 31 participants were excluded after MRI quality control, leaving 151 participants in the analysis (Table 1). Most participants were white (94.7%) and non-Hispanic (93.4%). The average (SD) BMI *Z*-score was 0.468 (0.95), and 30% of participants were categorized as either having overweight or obesity. The average (SD) caloric consumption of the standardized preload meal was 449 (171 kcal). Examining the distribution of *MC4R* rs571312 in our sample, 4.6% were highest-risk (AA) children (*N* = 7), 41.1% were heterozygotes (AC) children (*N* = 62), and 54.3% were homozygous low-risk (CC) participants (*N* = 82). Due to the limited number of participants in the highest-risk group, the *MC4R* AA and AC genotypes were collapsed into one category and analyzed with a dominant model (AA and AC vs. CC). In our sample, the distribution of *FTO* rs9939609 was 10.6% with the highest obesity risk (AA) (*N* = 16), 48.3% with moderate risk (AT) children (*N* = 73), and 41.1% with the lowest risk (TT) children (*N* = 62). The additive model of the *FTO* genotype (AA vs. AT vs. TT) was used in further analyses.

**Table 1:** Baseline characteristics of study participants (*N* = 151).

	Mean (SD) or *N* (%)
Age (years)	10.9 (1.16)
**Child biological sex**	
Male	86 (57.0%)
Female	65 (43.0%)
**Ethnicity**	
Non-Hispanic	141 (93.4%)
Hispanic	6 (4.0%)
Prefer not to answer	4 (2.6%)
**Race**	
White	143 (94.7%)
Non-White	8 (5.3%)
BMI *Z*-score	0.468 (0.953)
**BMI category**	
Underweight (<5th percentile)	1 (0.7%)
Healthy weight (5th to <85th percentile)	105 (79.5%)
Overweight (85th percentile to <95th percentile)	21 (13.9%)
Obese (≥95th percentile)	24 (15.9%)
**Household annual income**	
<$25,000	2 (1.3%)
$25,000–64,999	19 (12.6%)
$65,000–144,999	75 (49.7%)
$145,000–225,000	35 (23.2%)
>$225,000	16 (10.6%)
Prefer not to answer	4 (2.6%)
**Parent’s education level**	
High school graduate or GED	5 (3.3%)
Some post-high school, no degree	11 (7.3%)
Associates degree	8 (5.3%)
Bachelor’s degree	41 (27.2%)
Professional school or graduate school	85 (56.3%)
Missing	1 (0.7%)
**Physical activity (active for at least 60 min per day in the past 7 days)**	
No days	3 (2.0%)
1 day	4 (2.6%)
2–3 days	50 (33.1%)
4–5 days	61 (40.4%)
6–7 days	32 (21.2%)
Missing	1 (0.7%)
**European ancestry**	
European	136 (90.1%)
Non-European	15 (9.9%)
*MC4R* rs571312	
CC (0)	82 (54.3%)
AC (1)	62 (41.1%)
AA (2)	7 (4.6%)		Mean (SD) or *N* (%)
***FTO*_rs9939609**	
TT (0)	62 (41.1%)
AT (1)	73 (48.3%)
AA (2)	16 (10.6%)

### 3.1 ROI analyses

**Figure 2:**
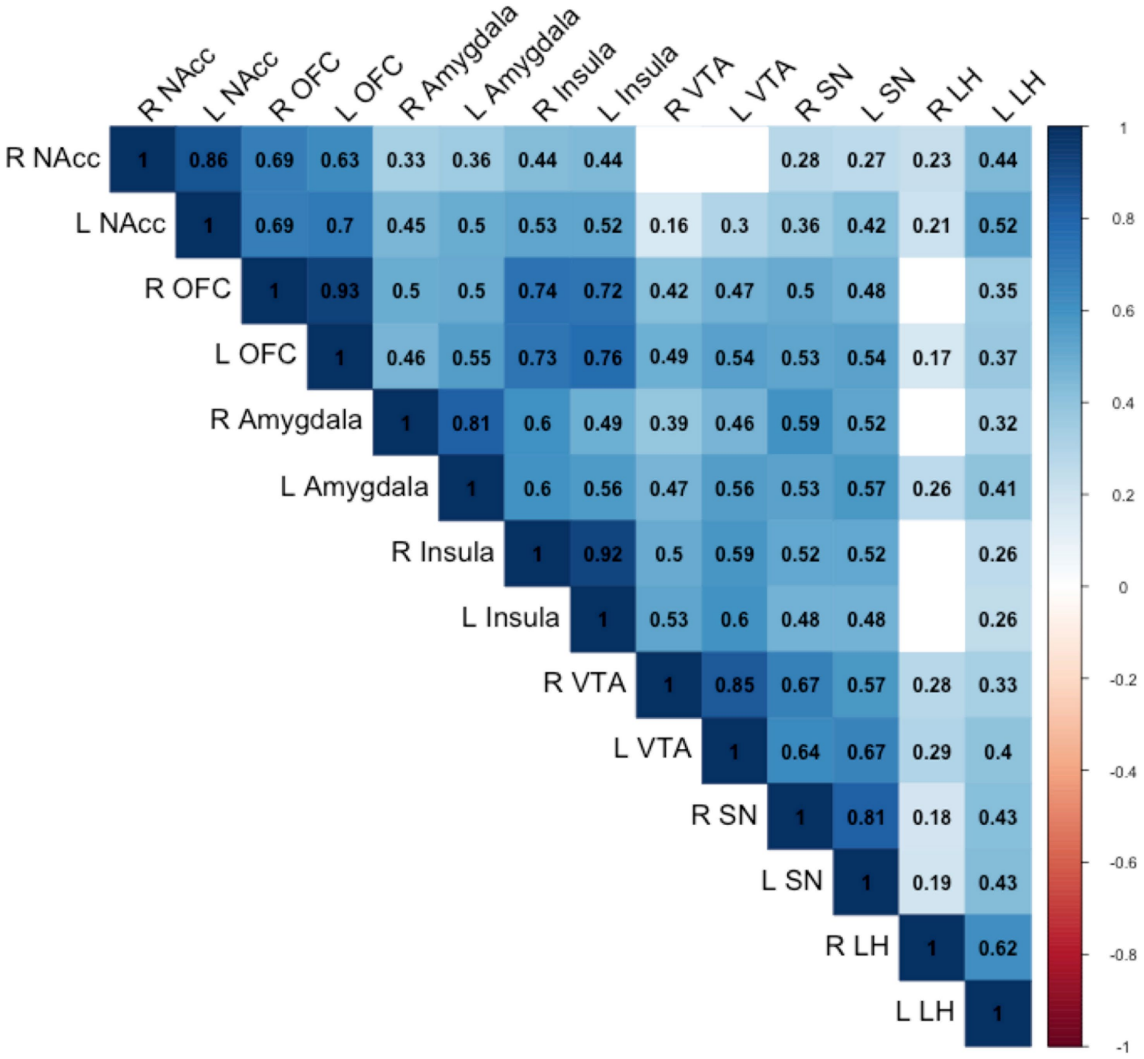
Pearson correlations between the reward regions are shown. Only correlations statistically significant at the *p* < 0.05 are numbered. NAcc, nucleus accumbens; OFC, orbitofrontal cortex; VTA, ventral tegmental area; SN, substantia nigra; LH, lateral hypothalamus.

**Figure 3:**
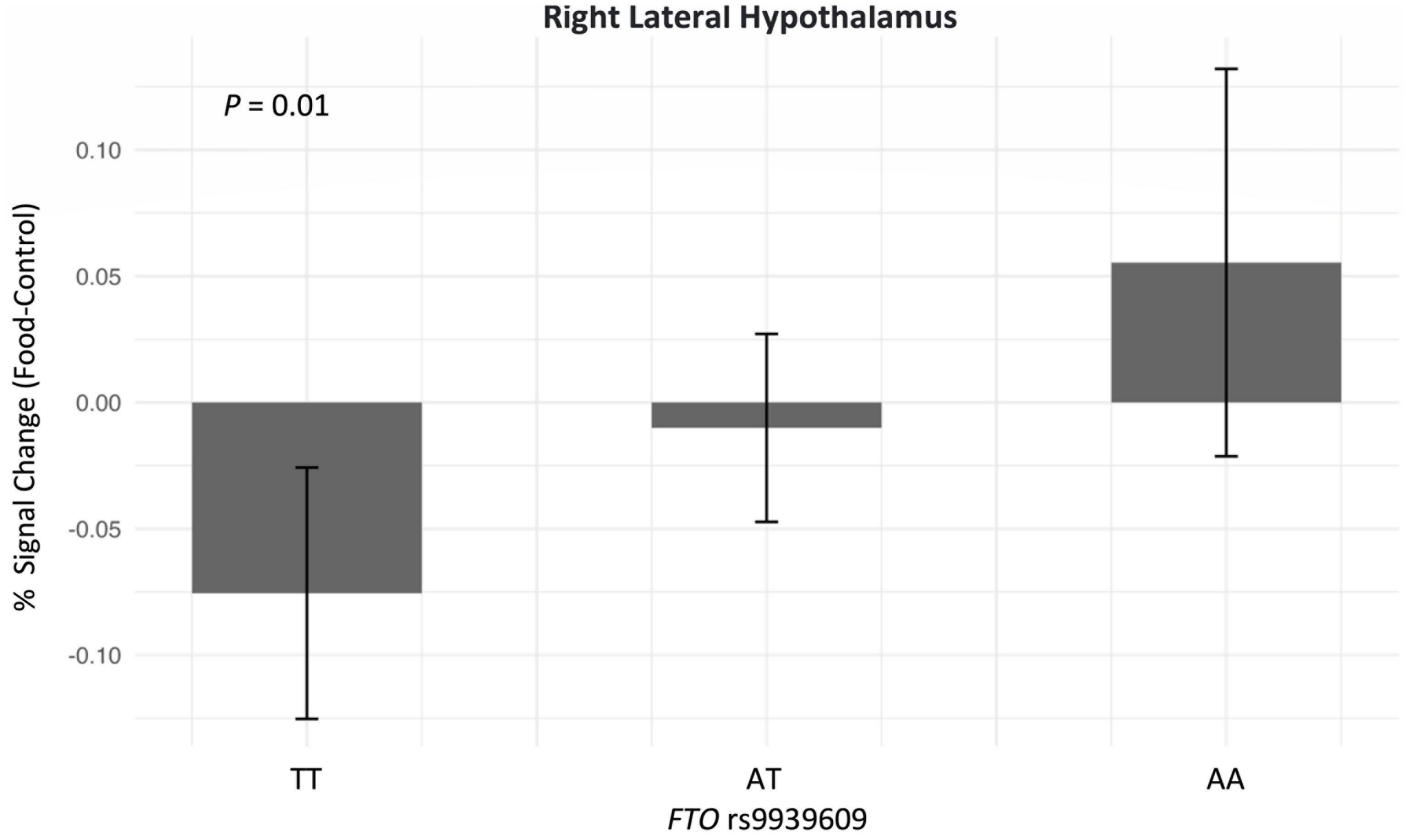
Adjusted association between *FTO* rs9939609 and neural response in the right lateral hypothalamus after eating a meal to satiety (*N* = 151). Adjusted linear regression model was conducted and adjusted for age, biological sex, BMI *Z*-score, satiety level post preload-meal, European ancestry, physical activity, and annual household income. The sample size of *FTO* rs9939609 for lowest-risk group (TT) is 62, heterozygotes (AT) is 73, and highest-risk group (AA) is 16. *FTO* rs9939609 was added as an additive model (TT vs. AT vs. AA).

The correlations between the responses of food > non-food contrast maps in 14 bilateral reward regions are shown in Figure 2. In general, all bilateral reward regions tested were positively and significantly correlated ranging from 0.93 for the OFC and 0.62 for the LH (Supplementary Figure S1). The right LH exhibited correlations with three other ROIs, and the bilateral VTA exhibited positive correlations with five ROIs. The ROI analysis examining the associations between each genetic exposure and neural response to food cues in the full cohort is presented in Table 2. Children with the *FTO* rs9939609 risk allele had a significantly higher food-related neural response in the right LH in models adjusted for covariates even after controlling for current adiposity (*t* = 2.6, *p* = 0.01) (Figure 3). However, the association did not remain significant after applying the FDR correction. The association between *FTO* rs9939609 risk alleles and food-related response in the left LH did not reach statistical significance (*t* = 1.7, *p* = 0.08). The *MC4R* genotype and Pediatric PRS were not significantly associated with the food-related reward reactivity in any of the explored ROIs (Table 2).

**Table 2:** Associations between genetic exposures and food-related response in the region-of-interest (ROI) after eating a meal to satiety (*N* = 151).

		Unadjusted models^a^		Adjusted model 2^a^^,^^b^	
	L/R	*t*-value	*p-*value	*t*-value	*p-*value
***FTO* rs9939609 (TT vs. AT vs. AA)**					
Nucleus accumbens	R	1.354	0.178	1.426	0.156
	L	1.081	0.281	1.017	0.311
Orbitofrontal cortex	R	0.035	0.972	−0.339	0.735
	L	0.076	0.940	−0.238	0.812
Amygdala	R	−0.892	0.374	−1.143	0.255
	L	−0.211	0.833	−0.291	0.772
Insula	R	0.368	0.713	−0.042	0.967
	L	0.669	0.505	0.340	0.734
Ventral tegmental area	R	−0.226	0.821	−0.116	0.908
	L	0.205	0.838	0.130	0.896
Substantia nigra	R	0.070	0.944	0.034	0.973
	L	0.036	0.971	−0.001	0.999
Lateral hypothalamus	R	1.958	0.052	**2.602**	**0.010**
	L	1.491	0.138	1.743	0.083
***MC4R* rs571312 (CC vs. AC + AA)**					
Nucleus accumbens	R	0.999	0.319	0.903	0.368
	L	0.260	0.795	−0.071	0.944
Orbitofrontal cortex	R	0.634	0.527	0.269	0.789
	L	0.731	0.466	0.265	0.792
Amygdala	R	−0.793	0.429	−0.837	0.404
	L	−0.221	0.825	−0.228	0.820
Insula	R	−0.614	0.540	−1.010	0.314
	L	0.045	0.964	−0.486	0.628
Ventral tegmental area	R	0.124	0.901	0.043	0.966
	L	0.119	0.905	−0.049	0.961
Substantia nigra	R	0.016	0.987	−0.126	0.900
	L	−0.247	0.806	−0.514	0.608
Lateral hypothalamus	R	1.201	0.232	1.120	0.265
	L	0.800	0.425	0.881	0.380
**Pediatric PRS *Z*-score**					
Nucleus accumbens	R	1.070	0.286	1.048	0.296
	L	1.057	0.292	1.051	0.295
Orbitofrontal cortex	R	1.088	0.278	1.005	0.317
	L	1.179	0.240	1.030	0.305
Amygdala	R	−0.392	0.696	−0.415	0.679
	L	−0.513	0.609	−0.475	0.636
Insula	R	0.748	0.456	0.558	0.577
	L	1.249	0.214	0.800	0.425
Ventral tegmental area	R	0.026	0.980	−0.267	0.790
	L	0.247	0.805	−0.106	0.916			Unadjusted models^a^	Adjusted model 2^a^^,^^b^		L/R	*t*-value	*p-*value	*t*-value	*p-*value
Substantia nigra	R	0.523	0.602	0.250	0.803
	L	−0.277	0.782	−0.446	0.656
Lateral hypothalamus	R	0.450	0.654	0.187	0.852
	L	0.984	0.326	0.897	0.371

a Bold values represent the statistical significance at *p*-value < 0.05.

b Covariates include BMI *Z*-score, age, sex, physical activity, annual household income, European ancestry, and satiety post-meal (%).

In the exploratory analysis that tested three additional adult obesity PRS associations, the 97 PRS with food cue-related activity in the left insula did not reach statistical significance (*t* = 1.9, *p* = 0.06) (Supplementary Table S2). In the sensitivity analyses of the PRS measures restricting the sample to participants with European ancestry (*N* = 136), the adult 97 and 557 PRS with food-related response in the LH did not reach statistical significance (*t* = 1.8, *p* = 0.08; *t* = 1.7, *p* = 0.08, respectively) (Supplementary Table S3).

No additional relationships between genetic factors and food-related responsivity in the whole brain analysis.

## 4 Discussion

In this study of 151 children aged 9–12 years old, we found that the genetic risk of obesity was associated with greater brain activation in response to food advertisements in the lateral hypothalamus after eating a meal to satiety. Specifically, each risk allele of the *FTO* rs9939609 genotype was associated with heightened food-related responsivity in the right lateral hypothalamus.

In the brain, *FTO* is highly expressed in the hypothalamus, a region involved in the regulation of central energy homeostasis to control body energy balance, energy expenditure, and food intake (78). Many studies have reported the connection between *FTO* SNPs and obesity-related traits such as BMI, body fat mass, waist circumference, hip circumference, and energy intake (79–81). In addition, *FTO* risk alleles have been related to fat or carbohydrate intake, reduced satiety, overeating, and loss of control over eating (39, 82, 83). The lateral hypothalamus, as part of the hypothalamus, receives a high level of melanocortinergic inputs from the arcuate nucleus of the hypothalamus. Animal studies have identified that the LH is specifically involved in food-seeking behaviors, reward behaviors, and autonomic function (84–86). Our study finding that *FTO* is related to greater food-related reward activity in the lateral hypothalamus when children are in a sated state highlights a potential biological mechanism that may mediate the association between genetic obesity risk and excess weight gain. As the understanding of the LH’s precise functions and mechanisms remains limited, further research is needed to understand if the higher food-related reward activity corresponds to cued non-homeostatic caloric intake in the post-prandial period.

In our previous study with an independent study cohort of the same age group, we observed a heightened reward response in the bilateral nucleus accumbens in response to food vs. non-food TV commercials among children with at least one *FTO* risk allele compared to those with no risk alleles (42). In that study, children were provided a light snack prior to imaging, rather than a full meal eaten to satiety. Together, these studies suggest that the *FTO* genotype is related to the heightened reward response to food cues in different brain regions at different states of satiety.

In our current study, we did not find significant neural reward reactivity to food cues associated with the *MC4R* genotype nor four PRS measures. Given the composite nature of PRS, they may have limited utility in clarifying the biological mechanisms underlying the genetic-reward activity relationship.

This study provides evidence that the *FTO* genotype is related to neural reward reactivity to food cues in children in the post-prandial period. Nevertheless, it is important to acknowledge the limitations of our study. Given the distinct role of the lateral hypothalamus in food-seeking and reward behaviors within the hypothalamus, we aimed to study this specific region. While we used a mask, future research could use manual segmentation of the lateral hypothalamus to potentially provide more accurate segmentation. Our sample predominantly consisted of individuals of a white, non-Hispanic population and relatively higher socioeconomic status. Additionally, the additive effect of the *MC4R* genotype was not explored due to the limited number the high-risk (AA) individuals. Due to limitations in sample size, the analysis did not extend to additional candidate genes associated with obesity. Future research should aim to explore the genetic effects on heightened neural reward responsivity in a larger and more diverse population, allowing for broader generalization of the findings.

## 5 Conclusion

Our findings indicate that some children possess a genetic predisposition towards heightened food-cue-related neural reward reactivity in the post-prandial period. Given the prevalence of extensive media exposure among children which often includes the promotion of a variety of unhealthy food products, it is crucial to understand the genetic influences on food-related neural responses in children and mitigate exposure that may contribute to excess consumption. Longitudinal studies are needed to understand whether this heightened reward response to food cues leads to greater cued consumption and, ultimately, to excess weight gain.

## Data Availability

The original contributions presented in the study are publicly available. This data can be found at: https://www.ncbi.nlm.nih.gov/gap/, phs003550.v1.

## Footnotes

^1^ http://freesurfer.net
