# Supplementary material for: Genetic associations with neural reward responsivity to food cues in children
Source: Front Nutr. 2024 Sep 25;11:1387514. doi: 10.3389/fnut.2024.1387514 (PMC11461328; doi:10.3389/fnut.2024.1387514)
Supplement: Supplementary file 1 [file Image_1.pdf]

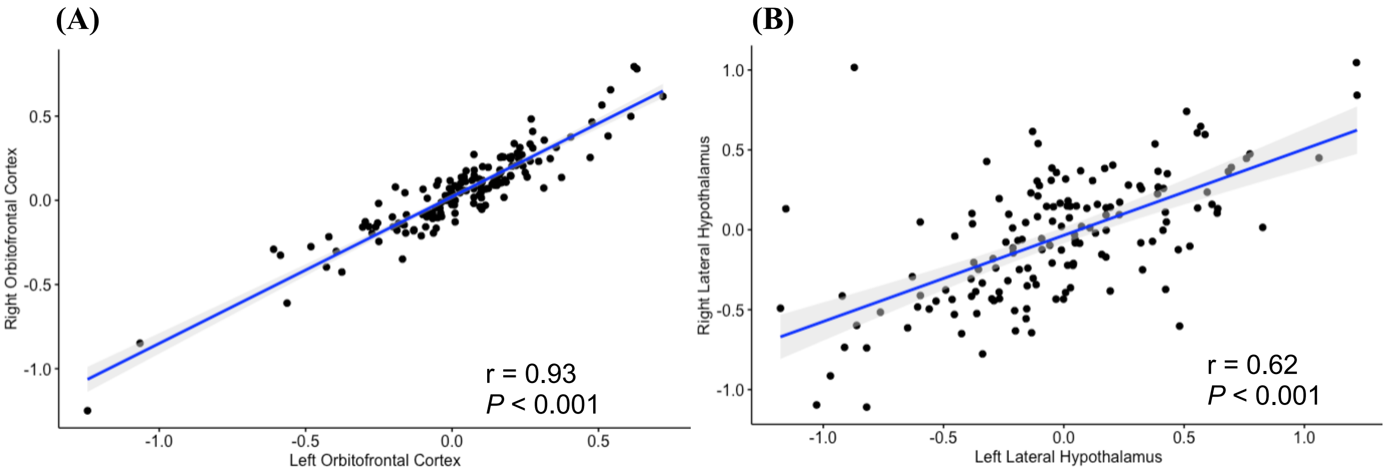

Supplementary Figure 1. Pearson's correlation tests for the left and right orbitofrontal cortex (A) and the left and right lateral hypothalamus (B).
