# Supplementary material for: Genetic associations with neural reward responsivity to food cues in children
Source: Front Nutr. 2024 Sep 25;11:1387514. doi: 10.3389/fnut.2024.1387514 (PMC11461328; doi:10.3389/fnut.2024.1387514)
Supplement: Supplementary file 2 [file Table_1.docx]

**Supplementary Table 1.** Distribution of PRS by European ancestry (N=151)

| **Characteristic** | European (N = 136) | Non-European (N = 15) | *P*-value |
| --- | --- | --- | --- |
| Pediatric PRS | 0.0151 (1.00) | -0.137 (1.02) | 0.591 |
| 97 PRS | 0.0368 (0.987) | -0.334 (1.09) | 0.224 |
| Adult PRS | 0.0378 (1.01) | -0.343 (0.861) | 0.128 |
| 2M PRS | -0.108 (0.954) | 0.982 (0.884) | <0.001 |
