# Supplementary material for: Genetic associations with neural reward responsivity to food cues in children
Source: Front Nutr. 2024 Sep 25;11:1387514. doi: 10.3389/fnut.2024.1387514 (PMC11461328; doi:10.3389/fnut.2024.1387514)
Supplement: Supplementary file 3 [file Table_2.docx]

**Supplementary Table 2.** Associations between PRS and food-related response in the region-of-interest (ROI) after eating a meal to satiety (N=151)

|  | | **Unadjusted Models^1^** | | **Adjusted Models^1,2^** | |
| --- | --- | --- | --- | --- | --- |
|  | *L/R* | t-value | *p-­*value | t-value | *p-­*value |
| **Adult 97 PRS** |  |  |  |  |  |
| Nucleus accumbens | R | 1.336 | 0.184 | 1.128 | 0.261 |
|  | L | 1.480 | 0.141 | 1.339 | 0.183 |
| Orbitofrontal cortex | R | 0.851 | 0.396 | 0.652 | 0.515 |
|  | L | 1.225 | 0.222 | 0.914 | 0.362 |
| Amygdala | R | -0.204 | 0.839 | -0.652 | 0.516 |
|  | L | 0.377 | 0.707 | 0.253 | 0.801 |
| Insula | R | 1.860 | 0.065 | 1.322 | 0.188 |
|  | L | **2.563** | **0.011** | 1.866 | 0.064 |
| Ventral Tegmental area | R | 1.901 | 0.059 | 1.364 | 0.175 |
|  | L | **1.996** | **0.048** | 1.383 | 0.169 |
| Substantia Nigra | R | **2.043** | **0.043** | 1.345 | 0.181 |
|  | L | 1.662 | 0.099 | 1.152 | 0.251 |
| Lateral Hypothalamus | R | 1.218 | 0.225 | 1.167 | 0.245 |
|  | L | 1.690 | 0.093 | 1.594 | 0.113 |
| **Adult 557 PRS** |  |  |  |  |  |
| Nucleus accumbens | R | 0.840 | 0.402 | 0.426 | 0.670 |
|  | L | 0.475 | 0.636 | 0.173 | 0.863 |
| Orbitofrontal cortex | R | 0.063 | 0.950 | -0.318 | 0.751 |
|  | L | 0.602 | 0.548 | 0.096 | 0.923 |
| Amygdala | R | 0.931 | 0.354 | 0.705 | 0.482 |
|  | L | 0.619 | 0.537 | 0.550 | 0.583 |
| Insula | R | 0.646 | 0.520 | 0.250 | 0.803 |
|  | L | 1.267 | 0.207 | 0.683 | 0.496 |
| Ventral Tegmental area | R | 0.988 | 0.325 | 0.512 | 0.609 |
|  | L | 1.435 | 0.153 | 1.110 | 0.269 |
| Substantia Nigra | R | 1.380 | 0.170 | 0.736 | 0.463 |
|  | L | 1.301 | 0.195 | 0.872 | 0.385 |
| Lateral Hypothalamus | R | 1.607 | 0.110 | 1.318 | 0.190 |
|  | L | 1.522 | 0.130 | 1.461 | 0.146 |
| **Adult 2M PRS** |  |  |  |  |  |
| Nucleus accumbens | R | -0.656 | 0.513 | -0.782 | 0.436 |
|  | L | -0.383 | 0.702 | -0.548 | 0.584 |
| Orbitofrontal cortex | R | **-2.093** | **0.038** | -1.517 | 0.131 |
|  | L | -1.232 | 0.220 | -0.856 | 0.393 |
| Amygdala | R | -1.434 | 0.154 | -1.336 | 0.184 |
|  | L | -1.044 | 0.298 | -1.035 | 0.302 |
| Insula | R | -0.864 | 0.389 | -0.652 | 0.515 |
|  | L | -0.171 | 0.865 | -0.205 | 0.838 |
| Ventral Tegmental area | R | 0.397 | 0.692 | -0.225 | 0.823 |
|  | L | -0.129 | 0.898 | -0.771 | 0.442 |
| Substantia Nigra | R | 0.249 | 0.804 | -0.023 | 0.982 |
|  | L | 0.784 | 0.434 | 0.335 | 0.738 |
| Lateral Hypothalamus | R | 0.717 | 0.474 | -0.335 | 0.738 |
|  | L | 0.445 | 0.657 | -0.176 | 0.860 |

^1^Bold values represent the statistical significance at p-value<0.05

^2^Covariates include BMI-z, age, sex, satiety post-meal (%), physical activity, annual household income, and European ancestry
