# Supplementary material for: Genetic associations with neural reward responsivity to food cues in children
Source: Front Nutr. 2024 Sep 25;11:1387514. doi: 10.3389/fnut.2024.1387514 (PMC11461328; doi:10.3389/fnut.2024.1387514)
Supplement: Supplementary file 4 [file Table_3.docx]

**Supplementary Table 3.** Associations between PRS and food-related response in the region-of-interest (ROI) after eating a meal to satiety, European ancestry participants only (N=136)

|  | | **Unadjusted Models^1^** | | **Adjusted Models^1,2^** | |
| --- | --- | --- | --- | --- | --- |
|  | *L/R* | t-value | *p-­*value | t-value | *p-­*value |
| **Pediatric PRS** |  |  |  |  |  |
| Nucleus accumbens | R | 0.542 | 0.589 | 0.493 | 0.623 |
|  | L | -0.069 | 0.945 | -0.039 | 0.969 |
| Orbitofrontal cortex | R | 0.358 | 0.721 | 0.405 | 0.686 |
|  | L | 0.401 | 0.689 | 0.387 | 0.699 |
| Amygdala | R | -1.581 | 0.116 | -1.492 | 0.138 |
|  | L | -1.587 | 0.115 | -1.504 | 0.135 |
| Insula | R | -0.575 | 0.566 | -0.732 | 0.465 |
|  | L | 0.126 | 0.900 | -0.355 | 0.723 |
| Ventral Tegmental area | R | -0.228 | 0.820 | -0.465 | 0.643 |
|  | L | -0.290 | 0.772 | -0.567 | 0.572 |
| Substantia Nigra | R | -0.252 | 0.801 | -0.511 | 0.610 |
|  | L | -1.252 | 0.213 | -1.293 | 0.198 |
| Lateral Hypothalamus | R | 0.988 | 0.325 | 0.618 | 0.538 |
|  | L | 0.637 | 0.525 | 0.549 | 0.584 |
| **Adult 97 PRS** |  |  |  |  |  |
| Nucleus accumbens | R | 1.321 | 0.189 | 1.099 | 0.274 |
|  | L | 1.333 | 0.185 | 1.214 | 0.227 |
| Orbitofrontal cortex | R | 0.401 | 0.689 | 0.502 | 0.617 |
|  | L | 0.693 | 0.489 | 0.632 | 0.528 |
| Amygdala | R | -0.392 | 0.696 | -0.539 | 0.591 |
|  | L | 0.102 | 0.919 | 0.149 | 0.881 |
| Insula | R | 1.334 | 0.185 | 1.060 | 0.291 |
|  | L | 1.864 | 0.065 | 1.330 | 0.186 |
| Ventral Tegmental area | R | 1.898 | 0.060 | 1.439 | 0.153 |
|  | L | 1.755 | 0.082 | 1.270 | 0.206 |
| Substantia Nigra | R | 1.748 | 0.083 | 1.199 | 0.233 |
|  | L | 1.265 | 0.208 | 0.882 | 0.379 |
| Lateral Hypothalamus | R | 1.790 | 0.076 | 1.385 | 0.169 |
|  | L | **1.998** | **0.048** | 1.816 | 0.072 |
| **Adult 557 PRS** |  |  |  |  |  |
| Nucleus accumbens | R | 0.901 | 0.369 | 0.431 | 0.667 |
|  | L | 0.471 | 0.638 | 0.115 | 0.909 |
| Orbitofrontal cortex | R | -0.187 | 0.852 | -0.341 | 0.734 |
|  | L | 0.363 | 0.717 | 0.024 | 0.981 |
| Amygdala | R | 1.005 | 0.317 | 1.049 | 0.296 |
|  | L | 0.319 | 0.750 | 0.425 | 0.672 |
| Insula | R | 0.281 | 0.779 | 0.084 | 0.933 |
|  | L | 0.749 | 0.455 | 0.295 | 0.768 |
| Ventral Tegmental area | R | 0.953 | 0.342 | 0.562 | 0.575 |
|  | L | 1.173 | 0.243 | 0.954 | 0.342 |
| Substantia Nigra | R | 1.416 | 0.159 | 0.889 | 0.375 |
|  | L | 1.189 | 0.237 | 0.812 | 0.418 |
| Lateral Hypothalamus | R | **1.995** | **0.048** | 1.334 | 0.185 |
|  | L | 1.909 | 0.058 | 1.748 | 0.083 |
| **Adult 2M PRS** |  |  |  |  |  |
| Nucleus accumbens | R | -0.424 | 0.672 | -0.398 | 0.691 |
|  | L | -0.220 | 0.826 | -0.240 | 0.811 |
| Orbitofrontal cortex | R | -1.278 | 0.203 | -0.974 | 0.332 |
|  | L | -0.575 | 0.566 | -0.428 | 0.669 |
| Amygdala | R | -0.590 | 0.556 | -0.468 | 0.641 |
|  | L | -0.677 | 0.499 | -0.476 | 0.635 |
| Insula | R | -0.132 | 0.895 | -0.465 | 0.642 |
|  | L | 0.400 | 0.690 | -0.260 | 0.795 |
| Ventral Tegmental area | R | 0.363 | 0.717 | 0.007 | 0.994 |
|  | L | 0.167 | 0.867 | -0.466 | 0.642 |
| Substantia Nigra | R | 0.583 | 0.561 | 0.290 | 0.772 |
|  | L | 0.777 | 0.439 | 0.453 | 0.651 |
| Lateral Hypothalamus | R | -0.118 | 0.906 | -0.656 | 0.513 |
|  | L | 0.207 | 0.837 | -0.021 | 0.983 |

^1^Bold values represent the statistical significance at p-value < 0.05

^2^Covariates include BMI-z, age, sex, satiety post-meal (%), physical activity, and annual household income
